# Usability of an independent arm modular robotic system in abdominal wall surgery: supraumbilical midline closure

**DOI:** 10.1590/0102-672020260000052e1981

**Published:** 2026-10-05

**Authors:** Bernardo Munoz SILES, Ricardo Zugaib ABDALLA, Ulysses RIBEIRO

**Affiliations:** 1 Universidade de São Paulo, Faculty of Medicine, University Hospital, Department of Gastroenterology – São Paulo (SP), Brazil.

**Keywords:** Robotic Surgical Procedures, Abdominal Wall, Hernia, Abdominal, Procedimentos Cirúrgicos Robóticos, Parede Abdominal, Hérnia Abdominal

## Abstract

**Background:**

Minimally invasive surgery, including robotic approaches, has proven effective in repairing various types of abdominal wall hernia defects, ranging from simple to complex cases. The Versius Surgical System® (CMR Surgical, Cambridge, United Kingdom) was developed to optimize the surgical experience and improve patient outcomes.

**Aims:**

This study aims to evaluate system applicability and its positioning and configuration within the operating room, using a preclinical *in vitro* model for supraumbilical midline closure.

**Methods:**

The study was conducted at the Teaching and Research Laboratory of Universidade de São Paulo University Hospital, using an in vitro synthetic latex abdominal wall model. For supraumbilical midline closure, the instrument units were positioned laterally (right and left), near the hypochondria, and the visualization unit was placed on the right side at the level of the pelvis. Surgical access points were created to allow for continuous suturing of an approximately 10 cm incision: one 10 mm suprapubic trocar and two 5 mm trocars placed bilaterally along the anterior axillary line at the level of the umbilicus.

**Results:**

The initial positioning of the robotic units allowed for an ergonomic configuration suitable for suturing. The continuous supraumbilical suture was performed using 2-0 barbed non-absorbable suture material, two needle drivers, and laparoscopic scissors, with stitch intervals of approximately 1 cm and moderate tension. Adequate closure of the hernia defect was achieved. The in vitro laboratory training phase totaled 20 hours. During this period, continuous suture was performed five times, with completion times ranging from 45 to 25 minutes.

**Conclusions:**

The Versius Surgical System® demonstrated full operational capability for supraumbilical midline closure in an inanimate model, without complications. Strategic positioning of the robotic arms allowed for adequate access and satisfactory docking times. Mastering the spatial positioning of the units is essential for optimizing the operative field and reducing non-productive time.

## ARTICLE HIGHLIGHTS

Operational viability: The Versius Surgical System® demonstrated full operational capability and safety for supraumbilical midline closure in an in vitro preclinical model.

Time optimization: A 20-hour laboratory training phase yielded a progressive reduction in continuous suturing time, decreasing from 45 to 25 minutes.

Ergonomics and access: The independent-arm modular platform provided suitable ergonomic configuration and precise access to the upper abdominal wall.

Spatial planning: Mastering the strategic positioning of robotic units and trocars is essential to optimize the operative field and reduce nonproductive time.

## Summary

**CENTRAL MESSAGE:**

Ventral hernia surgery has evolved significantly from large incisions involving multiple tissue layers to minimally invasive techniques. Laparoscopic surgery brought important advances—such as reduced surgical site infections, shorter recovery times, and shorter hospital stays—along with improvements in surgical technique. Robotic surgery has emerged as a solution to overcome the limitations of earlier methods, offering the surgeon greater precision, improved ergonomics, and enhanced comfort. While a single-module multi-arm system has dominated the market and the literature for over two decades, new robotic platforms featuring independent arm systems are now being introduced into surgical practice.

**PERSPECTIVES:**

The modular system (Versius Surgical System®) demonstrated full operational capability for supra-umbilical midline closure in an inanimate model, without complications. The strategic positioning of the robotic arms allowed for adequate access and satisfactory docking times. Mastering the spatial positioning of the units is essential for optimizing the operative field and reducing non-productive time. Future clinical studies are needed to validate these findings in actual patients.

## VISUAL ABSTRACT

**Figure:**
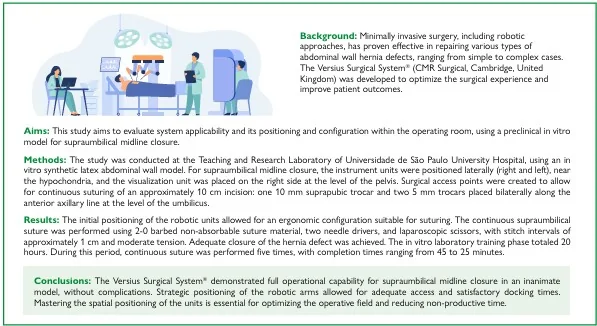

## RESUMO

**Racional:**

A cirurgia minimamente invasiva, incluindo abordagens robóticas, tem se mostrado eficaz na correção de diversos tipos de defeitos herniários da parede abdominal, tanto simples quanto complexos. O Versius Surgical System® (CMR Surgical, Cambridge, Reino Unido) foi desenvolvido para otimizar a experiência cirúrgica e os desfechos do paciente.

**Objetivos:**

Este estudo objetiva avaliar a aplicabilidade do sistema, seu posicionamento e disposição na sala de cirurgia, utilizando um modelo pré-clínico in vitro, para o fechamento da linha mediana supraumbilical.

**Métodos:**

O estudo foi conduzido no Laboratório de Ensino e Pesquisa do Hospital das Clínicas da Universidade de São Paulo, utilizando um modelo in vitro de parede abdominal sintética de látex. Para o fechamento da linha mediana supraumbilical, as unidades de instrumentos foram posicionadas lateralmente (direita e esquerda), próximas aos hipocôndrios, e a unidade de visualização, à direita, na altura da pelve. Foram criados acessos cirúrgicos permitindo sutura contínua de incisão de aproximadamente 10 cm: um trocáter de 10 mm suprapúbico e dois trocáteres de 5 mm bilateralmente na linha axilar anterior à altura da cicatriz umbilical.

**Resultados:**

O posicionamento inicial das unidades robóticas permitiu configuração ergonômica adequada para a sutura. A sutura contínua supraumbilical foi realizada com fio inabsorvível 2-0 unidirecional, utilizando dois porta-agulhas e tesoura videolaparoscópica, com intervalos de aproximadamente 1 cm entre pontos e tração média. O fechamento adequado do defeito herniário foi alcançado. A fase de treinamento em laboratório in vitro totalizou 20 horas. Durante este período, a sutura contínua foi executada cinco vezes, com uma variação no tempo de conclusão de 45 a 25 minutos.

**Conclusões:**

O Versius Surgical System demonstrou capacidade operacional completa para o fechamento da linha mediana supraumbilical em modelo inanimado, sem intercorrências. O posicionamento estratégico dos braços robóticos permitiu acesso adequado e tempos de acoplamento satisfatórios. O domínio do posicionamento espacial das unidades é essencial para otimização do campo operatório e redução de tempos improdutivos.

## INTRODUCTION

 Ventral hernia surgery has progressed significantly from large incisions involving multiple tissue layers to minimally invasive techniques. 

 Laparoscopic surgery has brought important advances, including reduced surgical site infections, shorter recovery times, shorter hospital stays, and better surgical techniques. However, conventional 2D laparoscopy has several limitations, including restricted depth perception, amplification of hand tremor, dependence on an assistant for camera control, limited instrument range of motion, and a steep learning curve, particularly in complex procedures involving manipulation of the abdominal wall (“surgical ceiling”)^4^. 

 Robotic surgery has emerged as a solution to overcome these limitations, offering greater precision, improved ergonomics, and enhanced surgeon comfort^9,10^. Although the Da Vinci Surgical System® (Intuitive Surgical, Sunnyvale, CA, USA) dominates the literature, with more than 20 years of clinical experience, new robotic platforms have emerged^6^. The Versius Surgical Robotic System® (CMR Surgical, Cambridge, UK) received European approval in 2019 and features a modular design, an open console, and instruments with a wide range of articulated movements^2^. Its four independent mobile units provide greater positioning flexibility and optimized access to the abdominal cavity, facilitating procedures such as supraumbilical midline closure. Compared with conventional laparoscopy, robotic surgery may provide improved visualization, greater dexterity, and enhanced control of surgical movements, facilitating complex procedures and potentially reducing the learning curve^5,7,8^. 

## METHODS

### Surgical technique

 The study was conducted at the Teaching and Research Laboratory of Universidade de São Paulo University Hospital, using an *in vitro* synthetic latex abdominal wall model. The Versius Surgical System® comprises a surgeon’s console featuring a 3D view enhanced by virtual reality goggles and joystick controls, along with three independent instrument units and a visualization unit. To close the supraumbilical midline incision, the instrument units were positioned laterally (right and left) near the hypochondria, while the visualization unit was placed on the right side at the level of the pelvis (Figure 1). Surgical access points were established to allow for continuous suturing of an approximately 10 cm incision: one 10 mm suprapubic trocar and two 5 mm trocars placed bilaterally along the anterior axillary line at the level of the umbilicus (Figure 2). 

**Figure 1:**
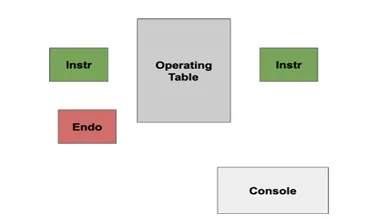
Laboratory configuration and positioning (personal archive).

**Figure 2:**
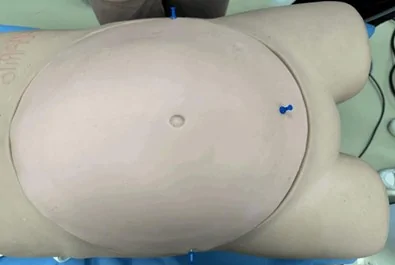
Silicone abdominal prosthesis (personal archive).

## RESULTS

 The initial positioning of the robotic units allowed for an appropriate ergonomic configuration for suturing. Continuous supraumbilical suturing was performed using a 2-0 unidirectional non-absorbable barbed suture, with two needle holders and laparoscopic scissors, maintaining approximately 1-cm intervals between stitches and moderate tension. Adequate closure of the hernia defect was achieved (Figures 3 and 4). 

**Figure 3:**
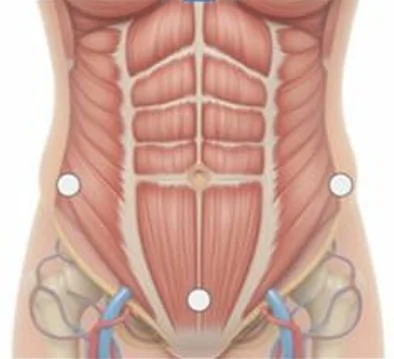
Trocar placement (personal archive).

**Figure 4:**
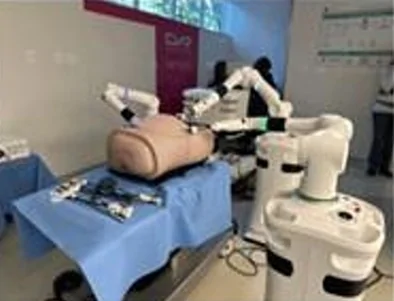
Positioning of the units for abdominal wall closure (personal archive).

 The in vitro laboratory training phase totaled 20 hours. During this period, continuous suture was performed five times, with completion times ranging from 45 to 25 minutes. This performance improvement was directly related to better positioning of the robotic arms and the surgeon’s adaptation to the platform^1^ (Figures 5 and 6). 

**Figure 5:**
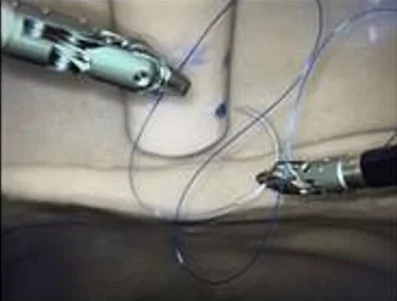
Beginning of suturing with non-absorbable suture (personal archive).

**Figure 6:**
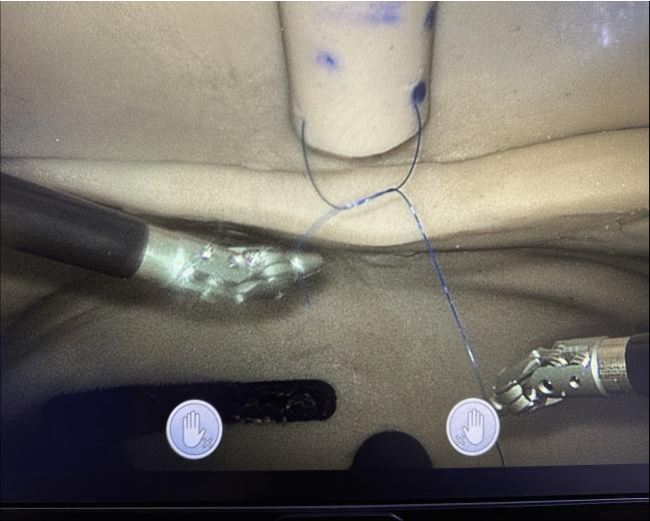
Continuous suture (personal archive).

 It is important to note that the study was conducted by a single operator, which represents a methodological limitation. Assistance tasks, such as suture cutting and repositioning of the robotic arms, were performed by the surgeon himself, which may have influenced the overall procedure time. 

## DISCUSSION

 The introduction of the Versius Surgical System® represents a significant advancement in general surgery and abdominal wall surgery. This study demonstrated the feasibility and safety of supraumbilical midline closure, with satisfactory technical outcomes despite the limitation of a single operator. The modular configuration of the robotic arms provides superior ergonomics and ceiling^5^. 

 The Versius Surgical System® enables the replication of conventional laparoscopic procedures while providing greater precision, fine tremor control, and high-definition threedimensional visualization. In addition, it improves surgeon comfort and ergonomics, which are crucial for safety and efficiency during complex procedures^10^. Compared with conventional laparoscopy, the Versius system offers advantages in terms of instrument mobility, dexterity, and stability, facilitating the adoption of minimally invasive surgery in mediumsized centers^3^. 

 However, limitations include the need for dedicated training and further evaluation of long-term clinical outcomes^1,9^. 

## CONCLUSIONS

 The Versius Surgical System® demonstrated full operational capability for supraumbilical midline closure in an inanimate model, without complications. Strategic positioning of the robotic arms provided adequate access and satisfactory docking times. Mastery of the spatial positioning of the robotic units is essential for optimizing the operative field and reducing nonproductive time. Further clinical studies are required to validate these findings in real patients. 

## Data Availability

The datasets generated and/or analyzed during the current study are available from the corresponding author upon reasonable request.
